# Senile Gangrene: Amputation of Thigh

**Published:** 1882-08

**Authors:** James B. Bell

**Affiliations:** Boston; Bureau of Surgical Therapeutics, I. H. A.


### SENILE GANGRENE—AMPUTATION OF THE THIGH.

JAMES B. BELL, M. D., BOSTON.

(*Bureau of Surgical Therapeutics, I. H. A.*)

I do not know how to make a better contribution to the subject of *surgical therapeutics*, than to report the following case in detail, as it shows very clearly the effects of suitable remedies, in attenuated doses, upon a very serious, painful and dangerous condition.

Mrs. G——, aged seventy-seven, of slender build, rather wiry constitution and active habits, but rather weak and dyspeptic for some years, had recently passed through a slow gastric fever under the care of my partner, Dr. William P. Wesselhoeft. October 15th, 1880, was seized suddenly in the early morning with acute burning pain in the left foot and leg. Dr. Wesselhoeft saw her at 8 A. M., and diagnosed embolism or thrombus of the left femoral artery, and probably gangrene. He gave Secale$^{cm}$ (Swan) in solution, every hour.

I saw her at 12 M., and found her in terrible distress, writhing upon the bed, and begging for relief or death. "Surely," some might reason, "here is a justifiable case for the use of Morphine." "A hopeless organic condition, causing such agony as is painful to witness, and with little possibility of the recovery of the patient. Beside *the books* say that only Morphine can relieve such cases."

Let us give the homœopathic remedy another chance, however, and perhaps the patient may yet be saved. The pains were *burning*, and extended through the whole leg and thigh, with a terrible bursting feeling as though the whole limb were very much too large. Leg cold and numb from the toes to the knee, purplish on the outer side. Patient very restless, and could bear very little covering. Gave Ars.$^{2c}$ (J.) in solution every hour.

The patient began to feel better very soon after the first dose, and in one hour was entirely comfortable.

It would certainly seem in this case as though Secale were better indicated than Arsenicum, especially with the aversion to covering, but perhaps the severely burning pains turned the scale in favor of the latter remedy.

*October 16th*, 10 A. M. Had a return of the pains twice last night for a short time. Leg much darker, purplish, and with a decided swelling above the knee. Ars.$^{8m}$ (J.) in solution every three hours. At 5 P. M. another dreadful attack. Restless, and in great distress. Says the leg is "burning up," that there is a "red-hot iron in it," and that it feels as though it were "being torn off." Great thirst, taking a teaspoonful every few minutes. This condition lasted for three hours until I saw her at 8 P. M.

This return of the pain would seem to prove that the former relief was not due, as some might think, to the progress of the death of the leg, but to the action of the remedy, which, now, however, ought to be changed for another. Guided by the burning sensations, which were now greater than ever, Cantharis was selected, and the 200th given in solution every fifteen minutes. She was entirely easy in one hour.

*October 23d.* Remains comfortable. Tongue cleaner. Some appetite. Pulse stronger, 82. No smell about the leg. Some signs of demarcation. More burning. Canth.$^{2c}$, one dose.

*October 25th.* Sleeping well. "Food tastes deliciously." Some black blisters on the dead part near the lining, and great tenderness of the adjacent living parts. Lachesis$^{cm}$ (Swan) in solution.

*October 30th.* Mummifying below the knee. Less soreness. Tongue more coated. More sleepless. Some burning heat in the thigh. Cantharis$^{cm}$ (Swan) in solution.

*November 1st.* Feeling better, but very wakeful before midnight. Puls.$^{16c}$ (J.) in solution.

*November 3d.* Had a good stool, aided by a warm-water enema, after twenty-five days without a movement. Sleeping well. Stronger than at any time.

*November 9th.* More nervous and sleepless again. Puls.$^{16c}$ (J.) one dose.

*November 13th.* Had a sore throat and some aphthæ in the mouth, but since a dose of Lachesis$^{cm}$, has gained again. Wake up a little wandering.

*November 18th.* Up to this time the patient had been well sustained by the treatment, aided by frequent feeding with nourishing food. The line of demarcation was now complete, the foot and leg were entirely mummified, and there had been at no time any offensive odor. Now also more signs of constitutional irritation began to appear; thrush, like that of infants, in patches upon the reddened mucous membrane of the mouth; a quicker pulse; more weakness and languor. The appetite was not lost, but the patient was too tired to eat. In view of these facts, and the possibility of restoration, amputation was advised. The patient and friends easily consented, and the operation was done.

*November 21st.* Thirty-seven days after the original seizure.

The operation chosen was by the anterior and posterior flap. The muscles were in a state of more or less fatty degeneration, and the vessels so calcified, that they would not hold a ligature, but were secured by including muscular and other tissue in the ligatures. Eleven were applied. The patient bore ether well and rallied without any shock. The femoral artery contained a solid plug above the incision.

*November 22d,* A. M. Had a very good night; is bright and strong. Pulse 112. Very thirsty. Slight pain. *Evening,* high fever. Wandering on waking. Has slept a good deal. Symphytum$^{cm}$ in solution.

*November 23d.* Rather weak and drowsy. Less fever. *Abdomen very tympanitic. Breathes with a puffing sound when asleep.* Cinchona$^{cm}$ in solution.

*November 24th.* A good night. Less tympanitic. Less fever. Wound looks very well.

*November 25th.* Dressed the wound and removed the sutures.

Very little or no union. Surface yellow and rather unhealthy looking. Some of the pus, bloody. Lachesis$^{cm}$ in solution.

*November 26th.* A good night. Tongue quite clean. Pulse 104. Suppuration thick and of good color. For the next eight days the condition was not very different. The pulse was often weak and intermittent, and there was considerable fever every evening, but a good amount of food was taken, and of nourishing character, administered in small quantities and at short intervals. The edge of the wound presented a red seam, and there were little sloughs at the corners and other portions, looking like false membranes. Drainage was carefully provided for, and a little Calendula used in all the washings and dressings, with a large oakum cushion over the stump all the time, and which was renewed every day. At this time (December 4th), because the tongue was red and dotted with patches of thick white fur, which also extended to the lips; and because of the symptom, " wound suppurates and does not heal well," Graphites$^{cm}$ was given. A marked, but gradual improvement in the whole condition took place, with separation of the slough, and slow but healthy granulation of the wound. About this time, in spite of every precaution, the sharp edge of the end of the femur pierced through the thin and atrophied anterior flap, cutting out a little round slough. The bone was not too long, but the psoas muscles were in a more or less constant state of contraction, often producing involuntary flexion of the stump, and no efficient way of antagonizing them could be devised. The bone soon began to granulate, however, and after a long time healed over. The patient recovered her usual health, and still lives, but, of course, is too thin and old to use crutches or to get about much.

This is the fifth consecutive amputation of the thigh under " Homœopathic Surgical Therapeutics," and without *Carbolic acid*, and all recovered.

I omitted to say that in addition to nourishing food, oatmeal, milk, game, some beef-tea, etc., every two hours, night and day, the patient also got a *quarter of a teaspoon of sherry*, diluted with water, every hour, for the greater part of the time.

